# Psychotropic medication profiles and future treatment risk among Northern Irish adults: a Latent Transition Analysis

**DOI:** 10.23889/ijpds.v9i5.1655

**Published:** 2024-09-18

**Authors:** Ron McDowell, Michael Rosato, Jamie Murphy, Gerry Leavey

**Affiliations:** 1 Ulster University, Coleraine, Northern Ireland

## Objective

Over recent decades, there has been a noticeable increase in the prescription of antidepressant medications, particularly among older adults. This study examines the relationship between health outcomes and patterns of antidepressant use among older adults in Northern Ireland (NI).

## Approach

Data linkage analysis of psychotropic prescriptions and household information. The ‘treatment episode’ was defined as the first identified period (>6 months) of psychotropic prescriptions preceded and followed by ≥ 12 months without psychotropics. Latent Transition Analysis was used to identify classes of psychotropic usage at initiation/discontinuation of treatment, with Cox regression used to estimate the likelihood of recommencing psychotropics.

## Results

We identified 157,836 patients, median age forty-five years (IQR 32,62) , 58.7% female, median exposure 1.5 years (IQR 1,2.5). Five latent classes of patients were identified in relation to received prescriptions: hypnotics, anxiolytics, antipsychotics, antidepressants and multiple psychotropics. The percentage of patients in the antidepressant class increased between initiation and discontinuation (from 64.2% to 67.1%) while that in the multiple psychotropics class fell (from 7.2% to 5.4%). 43% of patients who started treatment with multiple psychotropics had switched to antidepressants at discontinuation. These patients were significantly less likely to restart treatment than those who continued to receive multiple psychotropics (Hazard Ratio 0.83, 95%CI 0.76, 0.89) after adjusting for age at discontinuation, year of entry, deprivation, sex and duration of previous treatment.

## Conclusion

Continued receipt of multiple psychotropics is associated with an increased risk of future/ongoing treatment. This may reflect diagnostic uncertainly, complex disorder and discontinuation-associated side-effects.

## Implications

Researchers and practitioners alike should consider the diagnostic, health and treatment implications of these findings.

